# The wavelength of the ciliary beat in *Chlamydomonas* saturates at long ciliary lengths

**DOI:** 10.1016/j.bpj.2025.07.037

**Published:** 2025-08-05

**Authors:** Elijah H. Lee, Xiaoyi Ouyang, Jonathon Howard

**Affiliations:** 1 Department of Biomedical Engineering, Yale University, New Haven, Connecticut; 2 Department of Physics, Yale University, New Haven, Connecticut; 3 Department of Molecular Biophysics & Biochemistry, Yale University, New Haven, Connecticut

## Abstract

Cilia and flagella support bending waves that propagate along their lengths. In short cilia, such as those of the motile biflagellate green alga *Chlamydomonas reinhardtii*, the wavelength of the ciliary beat is approximately proportional to the length of the cilium. On the other hand, for the longer cilia of other organisms, such as sea urchin and mammalian sperm, the wavelength is shorter than the length, so that each cilium supports multiple wavelengths. These different wavelength/length ratios could be due to genetic or biochemical differences between species or due to length-dependent differences in the underlying physics of motility. To distinguish between these possibilities, we measured the beat wavelength in isolated, reactivated cilia from *Chlamydomonas* mutants in which ciliary length is mis-regulated, leading to cilia that are shorter or longer than the wild-type. This allowed us to probe the transition between short- and long-length behavior in a single organism rather than comparing different organisms. To test quantitatively the relationship between ciliary length and wavelength, we developed a Fourier-based estimator for the beat wavelength, accurate in the regime where the length is greater than half the wavelength. We confirmed that for shorter cilia, up to 15 *μ*m, the wavelength of the dynamic beat increased in proportion to ciliary length, as previously found. By contrast, in *lf4* mutants whose cilia are up to 25 *μ*m in length, the wavelength saturated at 15 *μ*m. Similar saturation was observed at both high and low ATP concentrations. These findings likely suggest that the physics of motility is important for determining the wavelength. We propose that the saturating wavelength is a trade-off between maximizing swimming speed (by making the wavelength as short as possible) and minimizing power consumption (by making the wavelength as long as possible).

## Significance

A well-known property of ciliated cells is that short cilia tend to have wavelengths similar to their lengths, whereas long cilia support multiple wavelengths. Because these observations come from different organisms or from different cells in the same organism, the wavelength differences may be due to different genes or gene expression. Alternatively, the differences may be due to length-dependent mechanical properties that underlie motility. To test the importance of length in the same species and cell type, we measured the wavelengths of cilia in wild-type and length-mis-regulated mutants of the unicellular alga *Chlamydomonas*. We found that the wavelength reached a maximum value as the length increased, suggesting that the mechanics of motility is important for determining the wavelength.

## Introduction

Cilia and flagella (hereafter collectively referred to as cilia) are thin, rod-like organelles that are ensheathed by the plasma membrane and protrude from the cell body. Cilia drive the motion of cells through fluids and drive fluids across the surfaces of cells. They play essential functions in locomotion, sensory reception, and signaling (1). The core structure within the cilium, known as the axoneme, is composed of nine pairs of doublet microtubules, a central pair of single microtubules, and a myriad of other proteins, including the axonemal dynein motor proteins, which generate the shear forces that slide adjacent doublets (2,3). Because sliding is constrained at the base and along the length, the dynein forces are converted into periodic bending motions that propagate along the axoneme (4,5,6). The mechanism of coordination of the dyneins in time and space, however, is not known.

Several mechanisms have been proposed for how the dyneins are coordinated. Most mathematical models of the ciliary beat, which originate in the classic work of Kenneth Machin (7), assume that periodic beating is due to a traveling wave of changing motor activity along the axoneme (though other models have been proposed (8)). To generate a self-sustained oscillation, the models assume that the dyneins partake in a feedback loop: they generate stresses and strains, which, in turn, are sensed by the motors, which alter their activities. Stresses include sliding forces parallel to adjacent doublets (9,10,11) and normal forces perpendicular to adjacent doublets (12). Strains include axonemal curvature (13) and the deformation of the axoneme’s circular cross section (14). Although models have established the plausibility of different feedback mechanisms in different organisms, it is still not known whether a single unifying mechanism can explain the beat patterns of all cilia.

Finding a unifying mechanism is challenging in part because cilia of different lengths often have different beat properties. For example, long cilia, such as those of sperm, often support several wavelengths (15,16), whereas short cilia, such as those found in ciliates and ciliated epithelia, usually support just a single wavelength (17,18). Does this mean that the dyneins in short and long cilia have different dynein-coordination mechanisms, as has been suggested by some models (14), or is there a universal coordination mechanism (11)? To answer this question, we need a cell type in one organism that can be induced to form short or long cilia: presumably the cilia will have the same biochemical properties, and any differences in beat waveform will be due to different mechanical properties arising from the different lengths.

The unicellular alga *Chlamydomonas reinhardtii* provides an experimental system to examine the length dependence of ciliary beating. Mutants have been isolated in which ciliary length control is mis-regulated, leading to cilia that are shorter or longer than the wild-type (19). Notably, there do not appear to be ultrastructural differences between the axonemes of these mutants (20,21). These findings allowed us to probe the transition between short- and long-length behavior in a single organism rather than comparing different organisms. We confirmed that in axonemes isolated and reactivated from shorter cilia, up to 15 *μ*m, the wavelength of the dynamic beat increased in proportion to ciliary length, as previously found. By contrast, for axonemes from *lf4* mutants, whose cilia grow up 30 *μ*m (20,22), the wavelength saturated at 15 *μ*m, showing that length does indeed change the beat shape. Because the hydrodynamic, frictional, and bending forces depend on ciliary length in different ways, our findings provide insight into the mechanics of beating (see discussion).

## Materials and methods

### Isolation and reactivation of axonemes

Axonemes were purified and reactivated from wild-type *C. reinhardtii* cells (CC-125 wild-type mt+ 137c, R.P. Levine via N.W. Gillham, 1968) and cells with ciliary length control mutants that have long (CC-4768 lf4-9 mt+ D12, L.W. Tam, 2013) and short (CC-2349 shf3-1851 mt+, J. Jarvik, 1989) cilia. All cells were purchased from the Chlamydomonas Resource Center at the University of Minnesota (United States). All chemicals were purchased from Sigma-Aldrich (Missouri, United States) unless stated otherwise.

Detailed methods can be found in Alper et al. (23). Here, we summarize the procedures. Strains were maintained on 2% agar plates, which were used to seed liquid cultures. Cells were precultured in tris-acetate-phosphate medium with additional phosphate (TAP+P) under continuous illumination and shaking for several days. Liquid preculture was used to seed larger 2 L TAP+P cultures, which were grown under 12-h light/dark illumination (24) with continuous air bubbling to a final density of 10^6^ cells per mL, checked via spectrophotometry at 750 nm for an optical density of at least 1.6 (25).

Before isolation, pH shock was used to deflagellate the cilia, synchronizing ciliary regrowth and enabling isolation of targeted lengths as described in Craige et al. (26) and Bottier et al. (17). After achieving the final desired density, cells were collected by centrifugation at 800 × *g* for 5 min and resuspended in 10% of the original volume of 10 mM HEPES buffer. The resuspension was titrated to pH 4.5 via dropwise addition of 0.5 N acetic acid under strong stirring. Addition was carried out as quickly as possible without overshooting the target pH and monitored using standard pH papers. After 60 s, the pH was quickly neutralized via drop-wise addition of 0.5 M potassium hydroxide. Cell bodies were centrifuged at 800 × *g* and diluted back to the original volume in fresh TAP+P. The synchronized culture was returned to growth conditions for the desired amount of time before proceeding with isolation.

Cilia were isolated using 5 mM dibucaine and then separated from the cell bodies by centrifugation using a 25% sucrose cushion (2400 × *g*). The cilia in the supernatant were demembranated in HMDEK (30 mM HEPES, 5 mM MgSO4, 1 mM DTT, 1 mM EGTA, 50 mM K-acetate) buffer augmented with 1% w/v IGEPAL and concentrated via centrifugation (32,000 × *g*). Demembranated axonemes were resuspended in 0.01% of the original culture volume in HMDEK (HMDEK augmented with 1% w/v polyethylene glycol) augmented with 1% w/v polyethylene glycol and 25% sucrose and stored at −80°C. All buffers after the wash step contained protease inhibitor (Pefabloc SC). Axonemes were thawed and kept on ice before reactivation.

Reactivation assays were performed in flow chambers with a depth of 100 *μ*m, constructed from easy-clean glass slides and coverslips (23) and double-sided sticky tape. Axonemes were diluted into HMDEKP reactivation buffer containing 1 mM ATP and an ATP regeneration system with 5 mM creatine phosphate and 5 units/L creatine kinase (27). The flow chamber was blocked using a 2 mg/mL casein solution (Sigma-Aldrich C7078) for 10 min. Excess reactivation buffer was used to wash the flow chamber before infusion of axonemes. The chamber was sealed using Valap (1:1:1 Vaseline, lanolin, and paraffin). Isolated cilia from all three strains exhibited high levels of reactivation (>90%). Reactivated axonemes tended to swim upward against the upper surface of the flow chamber where they are imaged. Cilia shorter than ∼5 *μ*m did not reactivate.

### Imaging axonemes

Axonemes were imaged by phase-contrast microscopy on a Zeiss Axioplan 2 upright microscope using a Zeiss Plan-NEOFLUAR 40× NA 1.3 Ph3 oil-immersion objective with a 1.6× optivar lens. Data were acquired using custom software written in LabView that controlled an EoSens MC1362 camera whose pixel size was 13.7 × 13.7 *μ*m (18). A stack of 1000 images was acquired for 1 s with a shutter time of 1 ms. The effective pixel size was 0.218 *μ*m, verified with a stage micrometer. Chambers were imaged for up to an hour.

### Tracking and analysis

**Figure 1:**
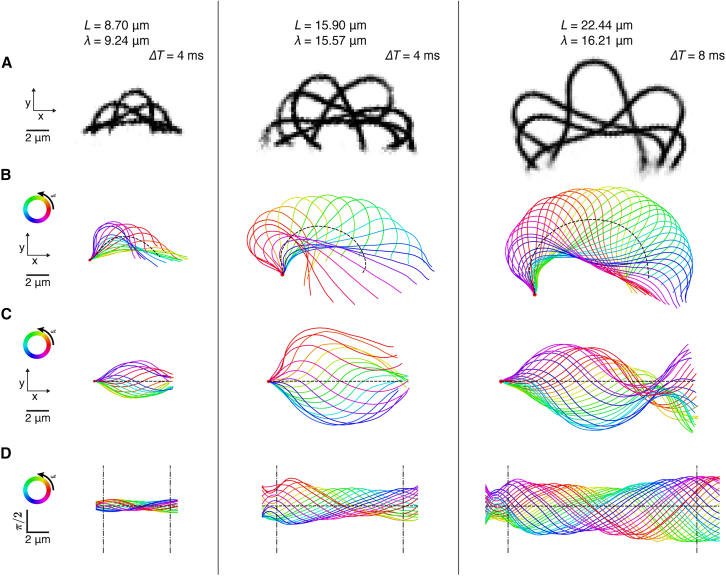
Shapes of three representative *lf4* axonemes from the different length regimes. The left and center columns show axonemes within the L≅λ regime, where *L* is length and *λ* is the wavelength. The right column shows an axoneme with a length beyond the wild-type length limit, with L>λ. (*A*) Processed phase-contrast images of a representative axoneme with L≅λ before inversion. Overlaid images were rearranged to show one full beat cycle, with imaging parameters described in the materials and methods. (*B*) Tracked shapes in Cartesian coordinates (x−y space) after subtraction of ciliary rotation (isolated axonemes swim in *circles*). The black dashed curve represents the static curvature (calculated from the time-averaged tangent angle). One beat cycle is shown with frames separated by 1 ms. (*C*) Cartesian coordinate representation of the dynamic component of the beat, reconstructed by subtracting the static curvature measured in angular ψ−s space (where ψ is the tangent angle and s is the arc length) so that the average curvature is zero (*black dashed line*). The “L=λ” relationship can be visualized here by observing the apparent “node” at length L (*left* and *center*). In the rightmost axoneme, the L>λ relation is evident because there is a “node” at s≅λ, and the start of a second simultaneous beat propagation is clearly visible at λ<s<L. (*D*) Tangent angle representation (ψ−s space) of the beat cycle after subtraction of the static component. Each line represents one frame, with the distance along the arc length s on the *x* axis and the tangent angle ψ on the *y* axis. Dashed-dotted black line denotes the middle 80% of the axoneme, which was used for wavelength calculation. The line representing the static component (the average tangent angle) has zero slope (i.e., no curvature). For a Figure360 author presentation of this figure, see https://doi.org/10.1016/j.bpj.2025.07.037.

Before digitization, images were inverted, and the mean intensity of all the images in a stack was subtracted from each image to increase the contrast. This initial image processing was done in Fiji (28). Sequential images of beating axonemes after preprocessing are overlayed in Fig. 1 *A*.

Axonemes were digitized using a custom Python script based on JFilament (29) to obtain the x−y coordinates of the centerline (https://github.com/OctopusProteins/cilia-analysis). The leading point of the swimming axoneme was placed at the basal end, where beat propagation begins. The average length, L, of each axoneme was then determined by integration. The tangent angle relative to the lab frame was computed for each point along the arc length for each frame.

Swimming axonemes rotate around a circle due to their nonzero curvature. To remove this rotation, we use the tangent angle (ψ) relative to the laboratory frame for the leading point as a function of time. This leading tangent angle oscillates around an increasing trend that was calculated via Locally Estimated Scatterplot Smoothing (LOESS) regression. The trend represents the rotation rate, which can be canceled via simple subtraction from the tangent angle representation in each frame. The rotation-subtracted shape was then transformed back into Cartesian coordinates (Fig. 1 *B*), showing an asymmetric beat.

The average tangent angle at each point is shown as the black dotted line in Fig. 1 *B* and is approximately circular. After subtracting the average tangent angle at each point along the arc length, we obtained the dynamic component of the beat in ψ−s space (Fig. 1 *D*). The transformation back into Cartesian coordinates is plotted in Fig. 1 *C*, which shows the symmetric dynamic beat in the x−y plane. The beat frequency was calculated via a discrete Fourier transform on the angular representation (ψ−s space) with quadratic interpolation of the power spectrum to find the peak. The wave velocity was calculated by tracking the zero-crossings in ψ(s).

### Wavelength determination via a Fourier-based estimator

The angular wavenumber of the dynamic beating component, $k_{0}=2\pi /\lambda_{0}$, where $\lambda_{0}$ is the wavelength, was estimated from the tangent-angle representation of the measured beat, ψ(s,t) (Fig. 1 *D*), using the Fourier-based estimator

$$\hat{k_{0}}=\max_{k}{\left\langle G_{\psi }\left(k,t\right)\right\rangle }_{t}\text{,}$$

where

$$G_{\psi }\left(k,t\right)={\left(\int_{-\infty }^{-\infty }\psi \left(s,t\right)\cos\left(ks\right)ds\right)}^{2}+{\left(\int_{-\infty }^{-\infty }\psi \left(s,t\right)\sin\left(ks\right)ds\right)}^{2}$$

is proportional to the power spectrum of ψ(s) at every time point, and ${\left\langle \right\rangle }_{t}$ is the average over all the frames. For each axoneme, the wavelength was determined by calculating the $G_{\psi }\left(k,t\right)$ numerically, averaging over each beat cycle, finding the maximum in *k*-space (in the range corresponding to a wavelength between a quarter and twice the axonemal length), and then averaging these maximum values over the 20–110 beat cycles recorded in the each 1 s stack.

In Appendix B, we show that for a sinusoidal traveling wave with wavelength approximately equal to the length (or shorter than the length), there is a small systematic error of <1%, which we ignore. We also show that the estimator is robust against added noise. As a third test for the validity of this procedure, we compared the estimator to the wavelength calculated as the wave velocity divided by the frequency (both measured as described above).

### Statistical testing of data obtained from different preparations

To test whether there were significant differences between the wavelengths of axonemes in different preparations, we counted the number of times, *n*, that the data from one preparation crossed the data in another preparation. We reasoned that if there were no differences, then we would expect that n=N/2, where *N* is the sample size and the the expected standard error of the mean is $\text{SE}=\sqrt{N}/2$. We therefore performed *t*-tests using t=|n−N/2|/SE with N/2 degrees of freedom. The Bonferroni correction was used when making multiple comparisons.

Because pairwise comparisons showed no significant differences, the data from the three *lf4* preparations were pooled. The two *wt* preparations also showed no statistical difference, and we therefore pooled the data from both preparations for subsequent analyses. The data are from a total of seven preparations (four *lf4*, including one used exclusively for the low ATP experiments, two *wt*, and one *shf3*). Error bars denote standard errors of the mean of the wavelength measurements calculated during each beat (20–110 beats in each 1 s of acquired data).

## Results

To measure the wavelengths of the axonemal beats of wild-type and mutant *C. reinhardtii*, we followed the isolation, demembranation, and reactivation procedures of Alper et al. (23) and Geyer et al. (18), as described in the materials and methods. Because the beat is asymmetric, the axonemes swim in circles and so can be imaged for long periods in the same field of view under the high-speed phase-contrast microscope. Swimming trajectories were collected for axonemes from wild-type cilia (lengths up to 12 *μ*m), the short-flagellar mutant *shf3* (lengths up to 12 *μ*m), and the long-flagellar mutant *lf4* (lengths up to 25 *μ*m) (22,30,31). To obtain a range of lengths in each strain, we harvested cilia after various growing times after deflagellation by pH shock (32). Motility was measured for cilia longer than 8 *μ*m. The motility of axonemes from mutant and wild-type cells was analyzed using a custom image-analysis pipeline, including a Fourier-based wavelength estimator (materials and methods).

### Measurement of the ciliary beat in Cartesian and angular spaces

After subtracting the rotation associated with the circular swimming paths, the raw images (Fig. 1 *A*) were digitized at 1 ms intervals (Fig. 1 *B*). The tangent angle was calculated at 0.218 *μ*m (pixel-wise) intervals along the arc length. The tangent angle was then decomposed into static and dynamic components (18), where the static component is defined as the average tangent angle over one beat cycle at each point along the axoneme. We found that the static component of the tangent angle was approximately linear, corresponding to a circular arc, shown as the dashed lines in Fig. 1 *B*. To isolate the dynamic component, we subtracted the static component in the angular representation space to yield the symmetric dynamic beat in Cartesian space (Fig. 1 *C*). The symmetric dynamic beat in angular space, ψ(s,t), is shown in Fig. 1 *D*.

### Dependence of the wavelength on length

The representation of the dynamic beat component in Cartesian space shows that the wavelength is approximately equal to the length for shorter axonemes but is shorter than the length for longer axonemes. For example, for the shorter axonemes in Fig. 1 *C*, the starting and ending points approximately superimpose at all times, forming “nodes” at both ends. In contrast, for the longer axoneme, the node is not at the end of the axoneme, implying that the wavelength is less than the length.

For each axoneme, we measured the wavelength using what we believe to be a novel Fourier-transform-based method, as described in the materials and methods section and Appendix A. In this method, we computed the power spectrum of each digitized waveform (in angular space) and estimated the wavelength as the maximum value of the average power spectrum for all 1000 images in the stack of images. In Appendix B, we show that the estimator is unbiased if the beat is a sinusoidal traveling wave with a wavelength equal to the length and that the bias is <1% when the wavelength is less than or equal to the length.

**Figure 2:**
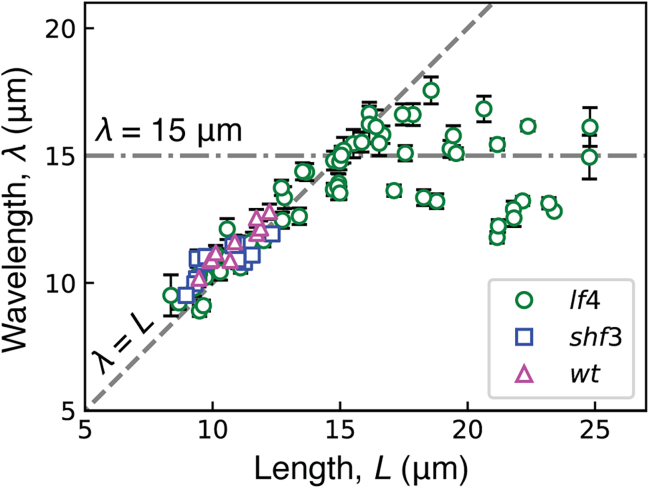
Dependence of wavelength on axoneme length. The wavelength measured using the estimator described in the materials and methods for *lf4*, wild-type, and *shf3* strains. For each axoneme, represented as a symbol, the error bar denotes standard error of the mean of wavelength measurements for each beat (between 20 and 110 beats). Data from three *lf4* preparations were pooled after a statistical test showed no significant differences (see materials and methods). Likewise, data from two wild-type preparations were pooled. Pairwise comparisons between the six preparations showed no significant differences in length dependence over their common length ranges (see materials and methods). The total numbers of axonemes were 60 (*lf4*), 10 (wild-type), and 12 (*shf3*).

For lengths between 8 and 15 *μ*m, the wavelength increased in proportion to the length (Fig. 2). This confirms earlier measurements (18). However, at a critical length near 15 *μ*m, the wavelength saturated at values within 13–17 *μ*m, despite further increases in length. Due to this saturation, multiple wavelengths are observed for axonemes longer than 15 *μ*m. There were no differences between *lf4*, wild-type, and *shf3* axonemes when compared over the same range of lengths (nonparametric statistic test; see the legend of Fig. 2).

### Length dependence of other beat properties

**Figure 3:**
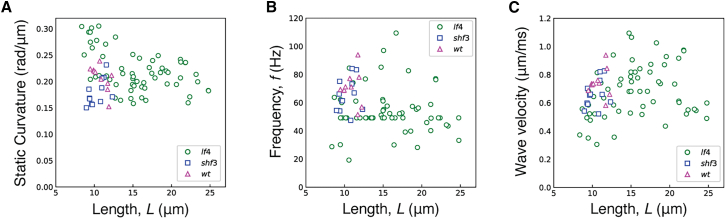
Dependence of beat parameters on length. (*A*) Static curvature. (*B*) Frequency. (*C*) Wave velocity. For each data set, linear regression was performed, and the correlation was deemed not significant if the slope, m, of the fit line (y=mx+b*)* was less than two standard errors from zero (the 95% confidence interval). (*A*) All data, m≈0.0003±0.0012(not significant), b≈0.201±0.018. *lf4* only, m≈−0.004±0.001 (significant). (*B*) m≈−0.638±0.444(notsignificant),b≈65.174±6.558 . (*C*) m≈0.006±0.005(notsignificant),b≈0.647±0.081.

We investigated the length dependence of other beat properties. There was no length dependence of static curvature when data from all axonemes were pooled (Fig. 3 *A*). To understand this result, we note that if static bending moments are applied to the ends of a uniform rod, then the curvature is independent of the rod’s length (curvature = moment/flexural rigidity). Therefore, if the curvature is independent of length (as observed), then the static moments are the same: this suggests that the number of moment generators at the ends (likely to be dyneins (13)) does not change as the length changes.

The beat frequency was also independent of axoneme length (Fig. 3 *B*). The wave propagation velocity depended on lengths up to 15μm (Fig. 3 *C*), as expected if the wavelength equals the wave velocity divided by the frequency.

### The wavelength is independent of the ATP concentration

**Figure 4:**
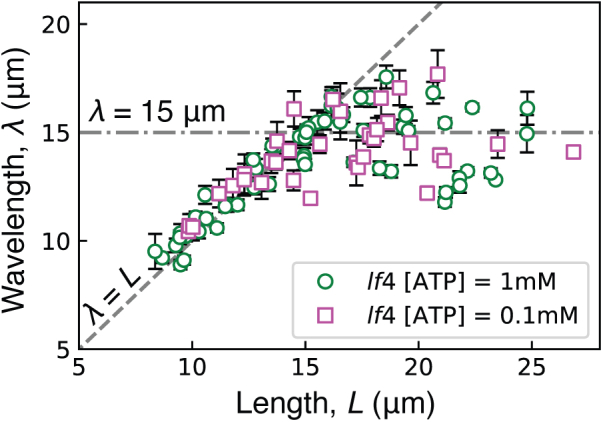
The wavelength does not depend on the ATP concentration. Plot of wavelength against length for *lf4* strain under standard (1 mM) ATP conditions and a low ATP (0.1 mM) condition. Error bars denote standard error on the mean of wavelength measurements from each beat (6–110 beats for each axoneme). A nonparametric zero-crossing test indicated that the curves were not significantly different (see materials and methods).

We also examined axonemal reactivation at low ATP concentrations (0.1 mM) where the beat frequency decreased roughly twofold, as previously observed (18). We found that despite the frequency changes, the length-wavelength relationship was unchanged (Fig. 4). This shows that the frequency and wavelength are uncoupled: the wavelength-length relationship is an intrinsic property of the axonemal structure and appears not to depend on the dynamics.

### Precision and robustness of the ciliary beat measurements

**Figure 5:**
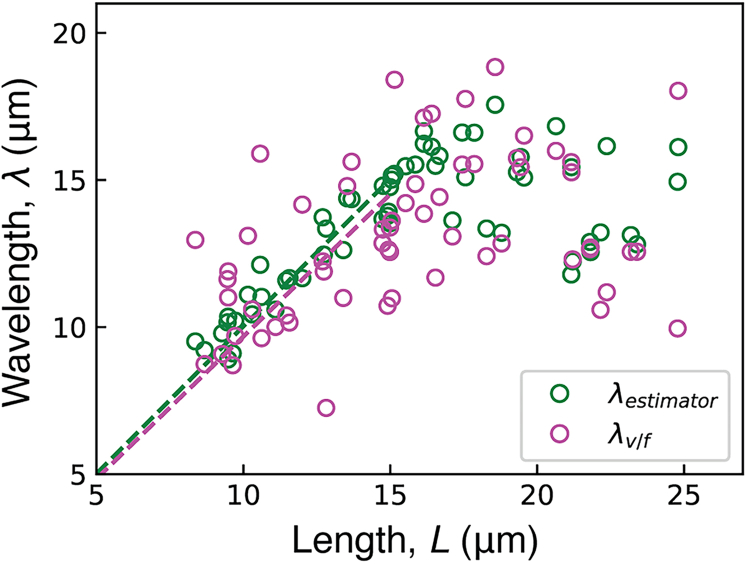
Comparison of the Fourier-based estimator with the estimation made from the wave velocity and the frequency. Data from *lf4* axonemes were used to compute wavelength using both the Fourier-based estimator as described (*green*), as well as from the wave velocity and frequency, where λ=v/f (*magenta*). Dashed lines show the fit from 8<L<15. For the estimator: m=1.002, root mean-square of residuals =0.772. For v/f: m=0.967, root mean-square of residuals =2.373.

We found that the estimator is more precise than the more conventional method of dividing the wave velocity by the frequency (see Fig. 5). Over the region 8μm≤L≤15μm, the Fourier method had a root-mean-squared residual (after a linear fit) of only one-third that of the conventional method (see legend of Fig. 5).

**Figure 6:**
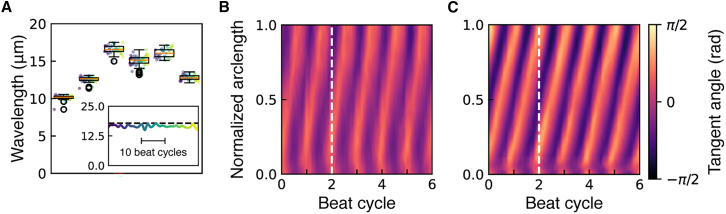
Robustness of the wavelength. (*A*) Boxplot of wavelength data for six representative *lf4* axonemes, with approximate lengths (*μ*m) from left to right: 9.5, 13.4, 17.9, 19.5, 22.7, and 23.4. Individual beat-wise wavelength measurements (in 1 s intervals) for each axoneme. Symbol colors denote time sequence (see *inset*). Boxplot shows the median (*orange line*), interquartile range (IQR; *box from Q1 to Q3*), and whiskers extending to the smallest and largest values within 1.5× IQR. Points beyond the whiskers are plotted as outliers. Inset: wavelength (in *μ*m) over 1 s for an axoneme of *L* = 17.9 *μ*m (*black dotted line*). Scale bar shows 10 beat cycles. Color denotes time sequence used in (*A*). (*B*) Kymograph of a representative axoneme with *L* = 8.7 *μ*m and *λ* = 9.24 *μ*m (in the regime where *L* = *λ*). White dashed line visualizes axoneme conformation at one point in time. (*C*) Kymograph of a representative axoneme with *L* = 22.37 *μ*m and *λ* = 16.15 *μ*m (in the regime where *L* > *λ*).

**Figure 7:**
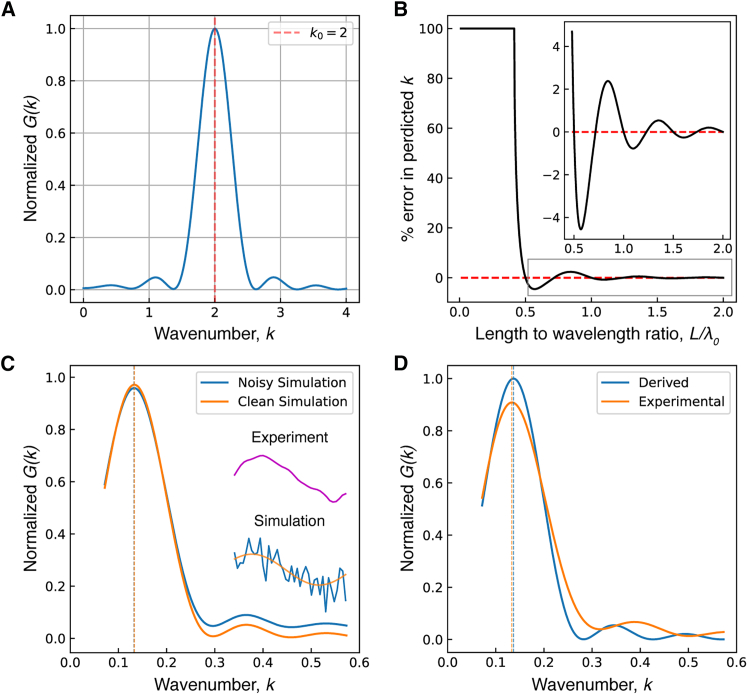
Fourier-based estimation of the angular wavenumber. (*A*) A normalized plot of the expected value of the $G_{\psi }\left(k\right)$ when $\lambda_{0}=L$. The red dashed line indicates the value of the true wavenumber, $k_{0}$, showing that the maximum value of $G_{\psi }\left(k\right)$ is an unbiased estimator of $k_{0}$. (*B*) Plot showing the error the estimator as a function of the ratio of length to the wavelength in the useful domain. The red dashed line indicates zero error. The inset shows a scaled view of the indicated area. (*C*) The estimator is robust to noise. The function was tested against a simulated, sinusoidal beat, both with and without strong uniformly distributed random noise (±50%). The calculated wavenumber is notated with a dashed line for each case. Inset shows representative experimental and simulated traces. (*D*) Comparison of the experimentally determined transform function G(k) (*orange*), averaged over all frames with the analytically derived solution (*blue*). The theoretical model is parameterized by the mean dominant wavenumber obtained from the experimental data, and the agreement in peak location (*dashed lines*) validates the model’s functional form.

The Fourier-based method shows that the wavelength is stable from one beat cycle to the next, and this is shown in Fig. 6 *A* (*inset*). Kymographs of two representative axonemes, one for each length regime, further emphasize the stability of the beating patterns. In Fig. 6 *B*, which depicts a representative axoneme in the wild-type length regime, the axoneme at one phase of the beat cycle encompasses one full range of potential tangent angles, as visualized by the white dashed line along which the tangent angle varies by 2π. This represents the propagation of a wavelength equal to the axonemal length. In contrast, Fig. 6 *C* shows a representative *lf4* axoneme longer than the wild-type. At one beat cycle (2 indicated by the *dashed line*), there are two positions along the axoneme (relative arc lengths of 0.15 and 0.85) where the maximum tangent angle is the same. This shows that, at any given time, more than one beat wavelength propagates down the axoneme. This confirms, using a different analysis, the results of Figs. 1 *C* (*right*) and 2. In addition to showing that the beat is robust, these data further support our finding that the longer axonemes support more than one wavelength. The accuracy of the Fourier-based wavelength estimator was further demonstrated by the simulations in Appendix B (Fig. 7).

## Discussion

Three observations reported in this paper provide insight into how the ciliary beat is generated. The first is that for lengths between 8 and 15 *μ*m, the wavelength is equal to the length (Fig. 3). This is true for axonemes with the same genotype (*lf4*) and so confirms and extends (over a wider range) earlier work showing equality when comparing different genotypes (18). Equality suggests that the beat-generating mechanism is a boundary value problem, in the sense that the boundary strongly influences the behavior, like the transverse vibration of a string in which the wave number is integrally related to the length. Support for the idea that the wavelength is constrained to be equal to the length (at short lengths) is that the correlation between wavelength and length is much tighter at short lengths than it is at longer lengths (Fig. 5), irrespective of how the wavelength is measured. The high variance at lengths ≥15μm suggests the lack of constraint when λ≥L.

There is some theoretical understanding for why the wavelength might equal the length. Geyer et al. (18) showed that for a curvature-controlled model, there are parameters that lead to analytic solutions to Machin’s equation that satisfy λ=L. The analytic solution was for short cilia, in the sense that the hydrodynamic forces are much smaller than the bending forces or $\text{Ma}=\left(f\xi_{⊥}\;\lambda^{4}\right)/\left(8\pi^{3}\kappa \right)\ll 1$, where Ma is Machin’s number, f is the beat frequency, $\xi_{⊥}$ is the hydrodynamic damping per unit length for motion of the axoneme perpendicular to its axis, and κ is the flexural rigidity (bending stiffness). For *Chlamydomonas*, Ma≅0.03 for L=15μm using the parameters from Geyer et al. (18). In other computational work (11), there are conditions in which a sliding-control model gives solutions with λ≅L (our solutions to this model). Thus, different models can account for the observation that λ=L.

The second observation is that there is a minimum length for a beat. Although we did not explore this part of the wavelength (length) curve in detail, a minimum length is consistent with earlier studies. For example, Bottier et al. (17) showed that a single cilium of length ≤4μm cannot rotate the cell body, suggesting that the beating pattern in this case is not a traveling wave but instead a standing wave, which, by the scallop theorem (33), cannot generate a propulsive force. Other studies show that ciliary forces are greatly attenuated at lengths below 4μm (34) and that pairs of cilia fail to synchronize below 5μm (35). A possible explanation for the failure to generate traveling waves at short lengths is that the power required to drive the beat increases as the wavelength decreases: overcoming internal damping between axonemes depends inversely on the wavelength squared and overcoming axonemal bending forces depends inversely on the wavelength to the fourth power (assuming sinusoidal beating patterns). If sliding or bending is limited by the power generated by the motors, then we expect there to be a minimum wavelength. Consistent with this argument, the amplitude of the beat is diminished when the wavelength is short (Fig. 1): the power also depends on the amplitude squared.

The third observation, the major result, is that the wavelength saturates at lengths greater than 15μm. Significantly, this is true for *lf4 Chlamydomonas* axonemes whose lengths differ only because of the amount of time they have been growing (and not because of genotype differences). Therefore, the wavelength dependence on length does not depend on genetic differences. To the best of our knowledge, this is the first time such an observation has been made in any species. Although it is possible that the saturation is due to biochemical differences between young and old axonemes, our observation that wild-type, *lf4*, and *shf3* axonemes of the same length (but different ages) all have the same wavelength argues against this. Instead, saturation is likely due to the different mechanics underlying beat generation in short and long axonemes. Furthermore, the existence of traveling waves at short lengths (L≅λ) and long lengths (L>λ) suggests that there is likely a single mechanism that generates the beat, irrespective of axoneme length.

The existence of a maximum beat wavelength is puzzling. As the wavelength increases, less and less power is needed to drive bending and shearing; the power to overcome hydrodynamic friction with the surrounding fluid is independent of wavelength (7). Therefore, a maximum wavelength cannot be due to a power limitation. On the other hand, the propulsive force per unit length generated by a straight axoneme undergoing a small-amplitude sinusoidal beat is $F/L=2\pi^{2}\left(\xi_{⊥}-\xi_{∥}\right)\left(fy_{0}^{2}\right)/\lambda$ (7). Thus, from a teleological point of view, the axoneme should have a wavelength as short as possible to maximize force production and the swimming speed, $v=F/\left(L\xi_{∥}\right)$. Although the breaststroke-like beat of *Chlamydomonas*’s two cilia has a more complex, asymmetric morphology, the argument is still valid: the propulsive force depends inversely on the beat wavelength. We therefore propose that the beat wavelength may be a trade-off between maximizing speed (make λ small) and minimizing power consumption (make λ large).

## Acknowledgments

This work was supported by the Yale College First-Year Summer Research Fellowship in the Sciences and Engineering and the Yale College Dean's Research Fellowship to E.H.L. and Yale’s Integrated Graduate Program in Physical and Engineering Biology to X.O. We thank Kai Zhang for laboratory support and Shengkai Li for ongoing discussions.

## Author contributions

E.H.L. performed experiments; E.H.L., X.O., and J.H. analyzed data; and E.H.L., X.O., and J.H. wrote the manuscript.

## Declaration of interests

The authors declare no competing interests.

## Appendix A: Analytic expression for the wavelength estimator of a sinusoid

For any function ψ(s),

$$G_{\psi }\left(k\right)\equiv {\left|\tilde{\psi }\right|}^{2}\equiv {\left|\int_{-\infty }^{-\infty }\psi \left(x\right)e^{-ikx}dx\right|}^{2}\equiv {\left(\int_{-\infty }^{-\infty }\psi \left(x\right)\cos\left(kx\right)dx\right)}^{2}+{\left(\int_{-\infty }^{-\infty }\psi \left(x\right)\sin\left(kx\right)dx\right)}^{2}$$

is proportional to the power spectrum of ψ. If ψ is sinusoidal with wavelength $\lambda_{0}$ over the interval [0,L],

$$\psi \left(x\right)=\sin\left(k_{0}x+\theta \right)\;x\in \left[0,L\right]k_{0}=2\pi /\lambda_{0}\text{,}$$

where θ∼U(0,2π) is a random phase uniformly distributed over [0,2π], then the Fourier transform of ψ is

$$\tilde{\psi }\left(k\right)=\int_{-\infty }^{\infty }R\left(\frac{x}{L}-\frac{1}{2}\right)\sin\left(k_{0}x+\theta \right)e^{-ikx}dx=\int_{-\infty }^{\infty }R\left(\frac{x}{L}\right)\sin\left(k_{0}x+\theta^{\prime }\right)e^{-ikx}dx\text{,}$$

where R is the rectangular function R(x)=1, $x\in \left[-\frac{1}{2},\frac{1}{2}\right]$, and the second equality follows from the change of variables $\theta^{\prime }=\theta +\frac{k_{0}L}{2}$, which is also ∼U(0,2π).

Using the three identities $F\left\{R\left(\frac{x}{L}\right)\right\}=\frac{L}{\pi }\text{sinc}\left(\frac{kL}{2}\right)$, where $\text{sinc}=\frac{\sin\;x}{x}$,

$$F\left\{h\left(k_{0}x+\theta \right)\right\}=e^{i\left(k/k_{0}\right)\theta }F\left\{h\left(k_{0}x\right)\right\},\text{and}$$

$$F\left\{\sin\left(k_{0}x\right)\right\}=\left(-i\pi \right)\left[\delta \left(k-k_{0}\right)-\delta \left(k+k_{0}\right)\right]\text{,}$$

we can express ψ as the convolution

$$\tilde{\psi }\left(k\right)=\frac{L}{\pi }\text{sinc}\left(\frac{kL}{2}\right)*\left\{e^{i\left(\frac{k}{k_{0}}\right)\theta }\left(-i\pi \right)\left[\delta \left(k-k_{0}\right)-\delta \left(k+k_{0}\right)\right]\right\}$$

$$=\left(-iL\right)\text{sinc}\left(\frac{kL}{2}\right)*\left\{e^{i\theta }\delta \left(k-k_{0}\right)-e^{-i\theta }\delta \left(k+k_{0}\right)\right\}\text{.}$$

Therefore,

$$G_{\psi }\left(k\right)=L^{2}{\left|e^{i\theta }\text{sinc}\left(\frac{\left(k-k_{0}\right)L}{2}\right)-e^{-i\theta }\text{sinc}\left(\frac{\left(k+k_{0}\right)L}{2}\right)\right|}^{2}\text{.}$$

Using $\frac{1}{2\pi }\int_{0}^{2\pi }{\left|e^{i\theta }a-e^{-i\theta }b\right|}^{2}d\theta =a^{2}+b^{2}$ for real a and b, the expectation of G over θ is

$$E_{\theta }\left\{G_{\psi }\left(k\right)\right\}=L^{2}\left\{{\text{sinc}}^{2}\left(\frac{\left(k-k_{0}\right)L}{2}\right)+{\text{sinc}}^{2}\left(\frac{\left(k+k_{0}\right)L}{2}\right)\right\}\text{.}$$

## Appendix B: Accuracy of the wavelength estimator

When $\lambda_{0}=L$, the expectation has a maximum at $k_{0}$, as shown in Fig. 7 *A*, and is unbiased. Provided that the length is greater than half the wavelength ($L/\lambda_{0}>0.5$), the estimator differs from the true wavelength by less than 5% (Fig. 7 *B*). When $L/\lambda_{0}\geq 1$, which is generally the case, the bias is <1%, and we do not correct for it.

The estimator is robust to noise. Fig. 7 *C* shows the effect of uniformly distributed noise on the estimator function with a simulated data set. The location of the maximum, the estimated wavenumber, was unchanged in the presence of strong (±50%) noise, despite slight differences in the shape of the function. Thus, our estimator performs better than peak detection or peak tracking-based methods, which are susceptible to noise.

As a further validation of the estimator, we compared the analytical solution (see Appendix A) with our numerical computation of the function using experimental data (Fig. 7 *D*). The experimental estimator was calculated from the tracked data and then averaged across all available frames. A key parameter in the analytical solution, the central wavenumber k, was set to the mean dominant wavenumber determined from the peak of the experimental G(k) for each frame. The plot in Fig. 7 *D* demonstrates an agreement between the shape of the averaged experimental transform function and the analytical model. Furthermore, the wavenumbers at which these respective functions reach their maxima, indicated by the dashed vertical lines, are closely aligned. This concordance validates that the chosen analytical form is a suitable representation of the transform function and that the experimentally derived mean wavenumber accurately parameterizes this model.

## Supporting material

Figure360. An author presentation of Fig. 1
